# Dietary total antioxidant capacity and frailty in Turkish community-dwelling and nursing home: cross-sectional study

**DOI:** 10.3389/fmed.2025.1577446

**Published:** 2025-04-04

**Authors:** Ömer Turan, Volkan Özkaya

**Affiliations:** ^1^Department of Nutrition and Dietetics, Graduate School of Health Sciences, Istanbul Medipol University, Istanbul, Türkiye; ^2^Department of Nutrition and Dietetics, Kutahya Health Sciences University School of Health Sciences, Kütahya, Türkiye

**Keywords:** older adult, dietary total antioxidant capacity, ADL, frailty, malnutrition, community, nursing home

## Abstract

**Background:**

This study examines the relationship between dietary total antioxidant capacity, frailty, and nutritional status in Turkish older adults living in the community and nursing homes.

**Methods:**

This study included 160 older adults (50% female) living in the community (*n* = 80) and a nursing home (*n* = 80). Anthropometric measurements were taken, and BMI was calculated. Demographic characteristics, nutritional status (MNA-SF: Mini Nutritional Assessment Short Form), frailty (FRAIL Scale), activities of daily living (Katz ADL), and three-day food consumption records were assessed. Dietary total antioxidant capacity was determined based on the three-day food consumption record.

**Results:**

The mean ages of the groups were similar (72.5 ± 6.0 and 72.2 ± 5.9 years). Nursing home residents had significantly higher rates of chronic disease (91.3%) and regular medication use (90.0%) (*p* < 0.05). Overweight was more prevalent among community dwellers (50.0%, *p* < 0.05), while obesity was more common in nursing home residents (26.2%, *p* > 0.05). Frail (32.5%) and pre-frail (40.0%) rates were higher in nursing home residents compared to elderly community dwellers (21.2 and 38.8%, respectively). Dependence ratios were similar between the groups (*p* > 0.05). Community-dwelling participants had a lower risk of malnutrition. While their daily carbohydrate intake was lower, nursing home residents had higher intakes of protein, fat, *ω*-3 fatty acids, fiber, vitamins (except vitamin E), and minerals. Frailty showed a strong negative correlation with Katz (*r* = −0.56, *p* < 0.001) and MNA-SF scores (*r* = −0.44, *p* < 0.001), while weak positive correlations were observed with TRAP, TEAC, and FRAP3 values. A negative correlation was observed between the residential setting and TORAC (*r* = −0.424, *p* < 0.001), TRAP (*r* = −0.190, *p* < 0.001), TEAC (*r* = −0.257, *p* < 0.001), and total VCEAC (*r* = −0.241, *p* = 0.002) values.

**Conclusion:**

Residential setting may affect nutrient intake, frailty, dietary total antioxidant capacity, and overall health in older adults.

## 1 Introduction

Due to medical, pharmacological, and technological advancements, the global population of individuals aged 60 and older is increasing significantly. Within the next 50 years, this population is projected to rise from 605 million to 2 billion (1). As of 2023, Türkiye has 8.72 million elderly individuals, accounting for 10.2% of the total population. Population projections indicate that the elderly proportion in Türkiye will rise to 25.6% by 2080 (2). During the aging process, all organs and systems undergo varying degrees of physiological changes. In addition to age-related physiological decline, older adults are at an increased risk of multiple chronic diseases, including osteoarthritis, dementia, hypertension, vision and hearing loss, osteoporosis, and cancer (3, 4). With age-related declines in physiological, functional, and cognitive reserves, along with reduced dynamic homeostasis, frailty becomes inevitable in the elderly population. Frailty is a biological syndrome characterized by a cumulative decline in the ability to maintain homeostasis due to age-related deterioration in physiological reserves and multiple physiological systems (4–7). It is a multidimensional condition influenced by factors such as age, gender (more common in women), body weight (both underweight and overweight), socioeconomic status, behavioral factors (smoking, alcohol consumption, diet, and physical activity), marital status, chronic health conditions, and psychosocial factors. While the global prevalence of frailty among individuals aged 65 and older is estimated to be around 10%, its reported prevalence ranges from 4.0 to 59.1% due to variations in frailty assessment criteria across epidemiological studies (8–12). Frailty is more prevalent among elderly individuals receiving care support or residing in nursing homes than among community-dwelling older adults, primarily due to advanced age, comorbidities, and decreased functional capacity. The prevalence of frail and frailty-prone elderly individuals has been reported as 52.3% in community dwellers, 73.2% in geriatric hospital patients, and 92.5% in nursing home residents (6, 9).

The identification of biological risk factors has significantly advanced the understanding of the mechanisms underlying frailty. In particular, chronic inflammation, increased oxidative stress, and mitochondrial dysfunction play key roles in the pathophysiological processes of frailty in older populations (13, 14). Dietary antioxidants can inhibit the production of reactive oxygen species and help mitigate oxidative DNA damage. Dietary total antioxidant capacity (DTAC) is a measure used to evaluate the cumulative, synergistic, and protective effects of all antioxidants present in the diet. DTAC is recognized as a useful measure for assessing the potential health benefits of consuming an antioxidant-rich diet and is defined as the total sum of all antioxidants obtained through dietary intake (15). Recent studies have reported that higher DTAC is associated with a lower risk of adverse health outcomes, including cardiovascular disease, type 2 diabetes, cancer-related mortality, and obesity (8, 16, 17). Similarly, higher DTAC has been linked to a lower prevalence of frailty and improved overall health (14, 18–20).

This study aims to investigate the relationship between frailty and dietary total antioxidant capacity in the Turkish elderly population and to assess the impact of residential setting on this association.

## 2 Materials and methods

### 2.1 Study design and participants

This cross-sectional study was conducted between March 2023 and March 2024 with Turkish individuals aged 65 and older, residing either in the Darülaceze Nursing Home in Istanbul or in the community-dwelling. A total of 160 participants were included in the study, selected through random sampling based on voluntary participation. The sample consisted of 40 women and 40 men from the nursing home and 40 women and 40 men from the community. The sample size was determined using the G*Power statistical analysis program, based on 85% power, an effect size of 0.5, and a significance level of *α* = 0.05. Ethics committee approval for the study was obtained from the Istanbul Medipol University Non-Interventional Clinical Research Ethics Committee (Approval No. E-10840098-604.01.01.01-1353, Date: 21/02/2023). The study was conducted in accordance with the principles of the Declaration of Helsinki, and informed consent was obtained from all participants.

The first part of the survey collected descriptive information, including age, gender, education level, physician-diagnosed diseases, and general health status. The second part assessed anthropometric measurements, dietary habits, daily nutrient intake, malnutrition, daily living activities, and frailty status. The data were collected through surveys conducted via face-to-face interviews with individuals who agreed to participate in the study.

**Figure 1:**
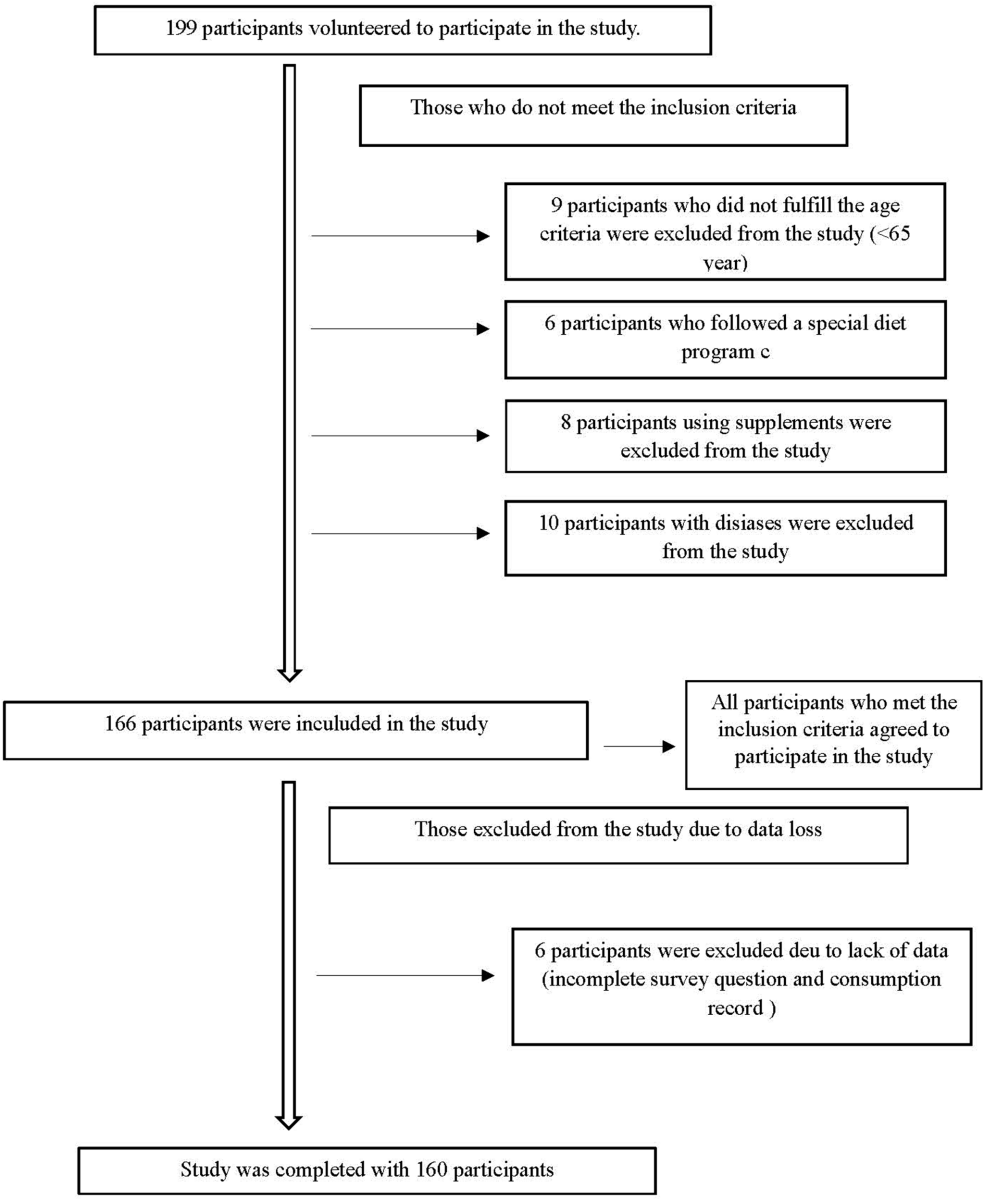
Participants recruitment flow chart.

Individuals with conditions affecting cognitive health (e.g., dementia, Alzheimer’s disease), depression, communication difficulties, cognitive or mental disorders, heart failure, cancer, chronic obstructive pulmonary disease, acute or chronic inflammatory diseases, as well as those receiving palliative care or who were bedridden, were excluded from the study. Additionally, individuals using nutritional supplements, following a special diet, or having a history of dysphagia were also excluded. The participant recruitment process is illustrated in the flow diagram (Figure 1).

### 2.2 Anthropometric measurements

Height measurements, accurate to 0.1 cm, were taken in centimeters using a portable stadiometer (Mesilife-13539) following the Frankfort plane. Body composition parameters, including body weight (kg), body fat percentage (%), fat mass (kg), and muscle mass (kg), were assessed using a Tanita MC-780 bioelectrical impedance analyzer. Body mass index (BMI) was calculated using the formula body weight (kg)/height (m^2^) and classified according to the World Health Organization (WHO) classification (21). Waist circumference (WC-cm) was measured midway between the lowest ribs and the iliac crest with the participant standing, arms at the sides, and feet together. Hip circumference (HC-cm) was measured at the widest part of the hips using a non-elastic tape measure with an accuracy of 0.1 cm while the participant was in a standing position.

### 2.3 Assessment of activities of daily living

Activities of daily living were evaluated using the Turkish version of the Katz Index of independence in activities of daily living (Katz ADL), originally developed by Katz et al. (22) and validated by Özkan Pehlivanoğlu et al. (23). The scale includes two response options for each function: dependent or independent. The maximum possible score is 6. A score of 6 on the Katz ADL indicates independence, scores between 3 and 5 indicate partial dependence, and a score of ≤2 reflects dependence.

### 2.4 Assessment of nutritional status

The Mini Nutritional Assessment-Short Form (MNA-SF) was used to evaluate the nutritional status of the participants (24). The Turkish validity and reliability of the MNA-SF, which is commonly used for screening nutritional deficiencies in older adults, were established by Sarikaya et al. (25). The scale includes questions on changes in food intake (loss of appetite, digestive issues, chewing or swallowing difficulties), weight loss, mobility, psychological stress or acute illness, neuropsychological problems, and BMI. Each question is scored between 0 and 3 points, with a maximum total score of 14 points. Based on the MNA-SF score, participants were classified into three categories: normal nutritional status (MNA-SF >11), at risk of malnutrition (MNA-SF: 8–11), and malnourished (MNA-SF <8).

### 2.5 Assessment of frailty

Frailty status was assessed using the Fatigue, Resistance, Ambulation, Illnesses, and Loss of Weight (FRAIL) scale, originally developed by Morley et al. (26) and validated for Turkish older adults by Hymabaccus et al. (27). The FRAIL scale scores range from 0 to 5, with 0 indicating the best condition and 5 the worst. Participants were categorized as robust (score = 0), pre-frail (score = 1–2), and frail (score = 3–5).

### 2.6 Assessment of food consumption: total antioxidant capacity and nutrients

To determine the participants’ energy and nutrient intake, 24-h dietary records were evaluated by the researchers over three consecutive days (two weekdays and one weekend day). A photographic atlas of food portion sizes, based on institutional standard recipes, was used to enhance the accuracy of dietary intake assessments. Food consumption data were analyzed using the Nutrition Information Systems Package (BeBiS, Ebispro for Windows, Germany; Turkish Version/BeBiS 9.0) with Standard Food Recipes (28). Energy and nutrient intakes were evaluated based on the Turkish Nutrition Guide-2022 (TÜBER-2022), which defines energy and nutrient requirements according to age and gender. The percentages of nutrient intake relative to daily recommendations were classified as <67% inadequate, 67–133% adequate, and > 133% excessive intake (29).

Participants’ food consumption records and dietary intake data were used to calculate DTAC. Two different methods were used to calculate Dietary Total Antioxidant Capacity (DTAC). First, the theoretical DTAC calculation was performed using, based on the National Food Composition Database values provided by the United States Department of Agriculture (Theoretical DTAC = ∑ (Antioxidant Content (mg/100 g)*Antioxidant Capacity (mg VCE/100 g)) (30, 31). After determining the antioxidant content of individual foods, the total daily antioxidant intake from all consumed foods was calculated. Mean DTAC values were expressed as vitamin C equivalent (VCE) mg/day. Since there is no national antioxidant database for foods consumed in Türkiye, the DTAC values used in this study were compiled from international databases. For foods not included in any database, values from foods with similar antioxidant content were used. The oxygen radical absorbance capacity (ORAC), trolox equivalent antioxidant capacity (TEAC), total radical-trapping antioxidant parameters (TRAP), and ferric-reducing ability of plasma (FRAP) methods were used to determine the total antioxidant capacity of foods (31–36).

### 2.7 Statistical analysis of the data

Statistical analysis was performed using SPSS version 22.0 (SPSS Inc., Chicago, IL, United States). In the data analysis, mean and standard deviation (mean ± SD) were used for continuous variables, while median, minimum, and maximum values were reported for specific characteristics. Categorical variables were presented as frequency (*n*) and percentage (%). Student’s *t*-test was used to compare the means of continuous variables between two independent groups. For comparisons among more than two independent groups, a one-way ANOVA was performed, and Tukey’s *post hoc* test was applied for pairwise comparisons when a significant difference was detected. The relationship between categorical variables was assessed using chi-square test statistics, while the relationship between continuous variables was evaluated using Pearson correlation analysis. Statistical significance was set at *p* < 0.05.

## 3 Results

Baseline characteristics of participants (*n* = 160), including the nursing home (*n* = 80, 50% female) and community (*n* = 80, 50% female) subgroups, are presented in Table 1. The mean age of individuals in the nursing home and community groups was 72.52 ± 6.09 and 72.28 ± 5.94 years, respectively. There was no statistically significant difference between the groups regarding educational status, smoking, and alcohol use (*p* > 0.05). However, the prevalence of chronic disease and regular medication use was 91.3 and 90.0%, respectively, in the nursing home group, compared to 78.7 and 73.7% in the community group, with these differences being statistically significant (*p* < 0.05). Body weight (kg), body fat percentage (%), total muscle mass (kg), and BMI (kg/m^2^) were 69.1 ± 12.3 kg, 30.6 ± 8.8%, 44.1 ± 7.8 kg, and 26.3 ± 4.3 kg/m^2^, respectively, in nursing home residents, and 68.6 ± 12.5 kg, 29.9 ± 7.8%, 44.5 ± 7.8 kg, and 26.2 ± 3.7 kg/m^2^ in community-dwelling participants (*p* > 0.05). Among the participants, 43.8% of nursing home residents and 35.0% of elderly community dwellers had a normal BMI. Notably, the prevalence of overweight was higher in community-dwelling participants (50.0%) compared to nursing home residents (28.7%) (*p* < 0.05). The obesity rates were 26.2% in nursing home residents and 13.7% in community dwellers. Although obesity was more prevalent among nursing home residents, this difference was not statistically significant (*p* > 0.05).

**Table 1:** Baseline characteristics of participants.

	Female		Male		Total	
Characteristic	Nursing home	Community	Nursing home	Community	Nursing home	Community
Gender *n* (%)	40 (50.0)	40 (50.0)	40 (50.0)	40 (50.0)	40 (50.0)	40 (50.0)
Education						
Illiterate	7 (17.5)	3 (7.5)	4 (10.0)	7 (17.5)	11 (13.8)	10 (12.5)
Primary school	19 (47.5)	20 (50.0)	20 (50.0)	18 (45.0)	39 (48.8)	38 (47.5)
Middle school	8 (20.0)	11 (27.5)	6 (15.0)	8 (20.0)	14 (17.5)	19 (23.7)
High school	5 (12.5)	4 (10.0)	6 (15.0)	5 (12.5)	11 (13.5)	9 (11.3)
University	1 (2.5)	2 (5.0)	4 (10.0)	2 (5.0)	5 (6.4)	4 (5.0)
Marital status						
Married	—	22 (55.0)^**^	—	18 (45.0)^**^	—	40 (50.0)^**^
Not married/single	40 (100.0)	18 (45.0)	40 (100.0)	22 (55.0)	80 (100.0)	40 (50.0)
Smoking						
Yes	7 (17.5)	9 (22.5)	23 (57.5)	17 (17.5)	30 (37.5)	26 (32.5)
No	33 (82.5)	31 (77.5)	17 (42.5)	23 (57.5)	50 (62.5)	54 (67.5)
Alcohol consumption						
Yes	—	1 (2.5)	4 (10.0)	3 (7.5)	4 (5.0)	4 (5.0)
No	40 (100.0)	39 (97.5)	36 (90.0)	37 (92.5)	76 (95.0)	76 (95.0)
Chronic disease						
Present	36 (90.0)	37 (92.5)	37 (92.5)	30 (75.0)	73 (91.3)	63 (78.7)^*^
Absent	4 (10.0)	3 (7.5)	3 (7.5)	10 (25.0)	7 (8.7)	17 (21.3)
Regular medication use						
Yes	36 (90.0)	32 (80.0)	36 (90.0)	27 (67.5)^*^	72 (90.0)	59 (73.7)^*^
No	4 (10.0)	8 (20.0)	4 (10.0)	13 (32.5)	8 (10.0)	21 (26.3)
Age (years) ( $\bar{X}\pm \mathrm{SD}$ )	72.0 ± 6.1	72.1 ± 5.2	73.5 ± 6.3	72.42 ± 6.6	72.5 ± 6.0	72.2 ± 5.94
Height (cm)	157.7 ± 7.8	156.0 ± 8.7	167.9 ± 6.4	166.6 ± 6.9	162.0 ± 9.1	161.3 ± 9.5
Body weight (kg)	66.0 ± 11.1	65.1 ± 12.7	73.1 ± 12.4	72.0 ± 11.5	69.1 ± 12.3	68.6 ± 12.5
BMI (kg/m^2^)	26.6 ± 4.9	26.5 ± 3.6	26.0 ± 4.7	25.9 ± 3.8	26.3 ± 4.3	26.2 ± 3.7
Waist circumference (cm)	100.3 ± 8.1	97.4 ± 14.8	102.8 ± 14.4	100.8 ± 12.8	100.3 ± 15.1	99.1 ± 13.9
Hip circumference (cm)	98.3 ± 12.0	94.4 ± 10.6	100.1 ± 9.5	96.6 ± 7.9	97.3 ± 10.2	95.5 ± 9.3^*^
Waist-to-hip ratio	1.0 ± 0.1	1.0 ± 0.06	1.02 ± 0.06	1.03 ± 0.06	1.01 ± 0.07	1.03 ± 0.07
Body fat percentage (%)	36.6 ± 7.2	34.6 ± 6.5	25.8 ± 8.9	25.3 ± 6.2	30.6 ± 8.8	29.9 ± 7.8
Total muscle mass (kg)	38.3 ± 4.6	38.9 ± 4.9	49.2 ± 6.4	50.2 ± 5.9	44.1 ± 7.8	44.5 ± 7.8
BMI category *n* (%)						
Underweight	—	—	1 (2.5)	1 (2.5)	1 (1.3)	1 (1.3)
Normal	19 (47.5)	13 (32.5)	16 (40.0)	15 (37.5)	35 (43.8)	28 (35.0)
Overweight	9 (22.5)	22 (55.0)^*^	14 (35.0)	18 (45.0)	23 (28.7)	40 (50.0)^*^
Obese	12 (30.0)	5 (12.5)	9 (22.5)	6 (15.0)	21 (26.2)	11 (13.7)

BMI, body mass index; ^*^p < 0.05 and ^**^p < 0.01.

Table 2 summarizes the quality of life, malnutrition, frailty, and dietary total antioxidant capacity of the participants by gender and residential setting. The mean Katz score was 4.91 ± 1.38 for nursing home residents and 4.97 ± 1.36 for community dwellers, with no statistically significant difference between the groups (*p* > 0.05). The dependence rates were similar between nursing home residents and community dwellers across genders. Among female participants, the proportion of those who were partially dependent was higher in nursing home residents (47.5%) compared to community dwellers (37.5%) (*p* < 0.05). The rate of independence was higher in men living in nursing homes (57.5%) compared to women living in the community (52.5%). Regardless of gender, the proportions of partially dependent and independent individuals were 37.5 and 53.7%, respectively, among community dwellers, and 41.2 and 50.0%, respectively, among nursing home residents. Among nursing home residents, the proportion of partially dependent individuals was higher, while the proportion of independent individuals was lower (*p* > 0.05). Among community dwellers, 42.5% (n = 34) had normal nutritional status, compared to 35.0% of nursing home residents (*p* > 0.05). The highest malnutrition rates were observed in men living in nursing homes (27.5%) and women living in the community (25.0%). Among elderly community dwellers, 40.0% were classified as robust, compared to 27.5% of nursing home residents. The highest frailty rates in both men and women were observed among nursing home residents. DTAC intake values were higher in nursing home residents compared to elderly community dwellers. In particular, the differences in TORAC, FRAP2, FRAP3, FRAP4, TEAC, and total VCEAC intakes were statistically significant (*p* < 0.05).

**Table 2:** Quality of life, malnutrition, frailty, and DTAC by gender and residential setting.

Determinants	Female		Male		Total	
	Nursing home	Community	Nursing home	Community	Nursing home	Community
KATZ Index ( $\bar{X}\pm \mathrm{SD}$ )	4.67 ± 1.47	4.90 ± 1.37	5.05 ± 1.31	5.05 ± 1.37	4.91 ± 1.38	4.97 ± 1.36
KATZ Index category *n* (%)						
Dependent	4 (10.0)	4 (10.0)	3 (7.5)	3 (7.5)	7 (8.8)	7 (8.8)
Partially dependent	19 (47.5)	15 (37.5)	14 (35.0)	15 (37.5)	33 (41.2)	30 (37.5)
Independent	17 (42.5)	21 (52.5)	23 (57.5)	22 (55.0)	40 (50.0)	43 (53.7)
MNA-SF score ( $\bar{X}\pm \mathrm{SD}$ )	10.15 ± 2.52	9.95 ± 2.66	9.90 ± 3.20	10.92 ± 2.31	10.23 ± 2.70	10.43 ± 2.53
MNA-SF score category *n* (%)						
Malnourished	6 (15.0)	10 (25.0)	11 (27.5)	4 (10.0)	17 (21.3)	14 (17.5)
At risk	22 (55.0)	14 (35.0)	13 (32.5)	18 (45.0)	35 (43.7)	32 (40.0)
Normal	12 (30.0)	16 (40.0)	16 (40.0)	18 (45.0)	28 (35.0)	34 (42.5)
FRAIL scale ( $\bar{X}\pm \mathrm{SD}$ )	2.05 ± 1.51	1.80 ± 1.77	1.65 ± 1.62	1.57 ± 1.67	1.76 ± 1.64	1.68 ± 1.71
FRAIL scale score category *n* (%)						
Non-frail	8 (20.0)	16 (40.0)	14 (35.0)	16 (40.0)	22 (27.5)	32 (40.0)
Prefrailty	16 (40.0)	10 (25.0)	13 (32.5)	13 (32.5)	29 (36.25)	23 (28.7)
Frailty	16 (40.0)	14 (35.0)	13 (32.5)	11 (27.5)	29 (36.25)	25 (31.3)
DTAC ( $\bar{X}\pm \mathrm{SD}$ )						
TORAC (μmol TE/100 g)	15180.8 ± 3718.7	13372.2 ± 4717.2^*^	17391.2 ± 3825.7	12714.3 ± 3701.5^**^	17391.2 ± 3825.7	12714.3 ± 3701.5^*^
TRAP (mmol TE/100 g)	2.82 ± 1.02	2.94 ± 1.77	3.17 ± 1.78	2.61 ± 1.63^*^	3.01 ± 1.44	2.78 ± 1.59
FRAP1 (mmol Fe/100 g)	3.64 ± 1.41	4.63 ± 2.90	4.01 ± 1.38	3.94 ± 2.29	4.01 ± 1.38	3.94 ± 2.29
FRAP2 (mmol Fe/100 g)	4.18 ± 2.07	2.90 ± 1.09^*^	4.34 ± 2.06	2.85 ± 1.45^**^	4.34 ± 2.06	2.85 ± 1.45^*^
FRAP3 (mmol Fe/100 g)	9.74 ± 5.91	7.09 ± 4.00^*^	10.41 ± 5.87	6.46 ± 4.17^**^	10.41 ± 5.87	6.46 ± 4.17^*^
FRAP4 (mmol Fe/100 g)	7.73 ± 6.26	3.69 ± 2.12^**^	7.17 ± 5.26	3.84 ± 4.08^**^	7.17 ± 5.26	3.84 ± 4.08^*^
TEAC (mmol TE/100 g)	3.76 ± 1.92	3.13 ± 1.53	3.88 ± 1.85	2.84 ± 1.60^*^	3.88 ± 1.85	2.84 ± 1.60^*^
Total VCEAC	38047.2 ± 15717.0	30942.0 ± 14956.6^*^	36556.7 ± 23259.4	25171.0 ± 12150.4^*^	36556.7 ± 23259.4	25171.0 ± 12150.4^*^

Katz, Katz Index of Independence in Activities of Daily Living; MNA-SF, Mini Nutritional Assessment Short Form; FRAIL, Fatigue, Resistance Ambulation Illnesses & Loss of Weight; DTAC, dietary total antioxidant capacity; TORAC, total oxygen radical absorbance capacity; TRAP, total radical-trapping antioxidant parameters; FRAP, ferric ion reducing antioxidant potential; TEAC, trolox equivalent antioxidant capacity; VCEAC, vitamin C mg/100 g equivalent; ^*^p < 0.05 and ^**^p < 0.01.

Participants’ energy, macro and micronutrient intakes, and percentages meeting recommendations are summarized in Table 3. No significant difference was found in inadequate energy (kcal) intake based on gender and place of residence. Daily carbohydrate (g) intake was lower in nursing home residents compared to elderly community dwellers, while protein (g) and fat (g) intakes were higher in nursing home residents. A statistically significant difference was found between nursing home residents and community-dwelling participants in terms of daily protein intake (g) and the percentage contribution of protein to energy (*p* < 0.01). A significant difference was found in the daily intake of *ω*-3 fatty acids between elderly community dwellers (1.52 ± 0.91 g) and nursing home residents (1.73 ± 0.7 g) (*p* < 0.01). The daily fiber intake was significantly higher in nursing home residents (29.9 ± 6.0 g) compared to elderly community dwellers (22.9 ± 6.2 g) (*p* < 0.01). Both men (29.1 ± 5.4 g) and women (30.8 ± 6.6 g) living in nursing homes had significantly higher daily fiber intake than their community-dwelling counterparts (women: 22.2 ± 5.6 g, men: 23.6 ± 6.8 g). The daily vitamin C intake of individuals living in the community (146.3 ± 55.7 mg) was lower than that of those living in nursing homes (153.9 ± 51.9 mg) (*p* > 0.05). Although the daily vitamin E intake was higher in community dwellers (12.2 ± 5.2 μg) than in nursing home residents (11.0 ± 3.3 μg), the difference was not statistically significant (*p* > 0.05). The daily intakes of vitamins B1, B2, B6, B12, and folate were significantly lower in community-dwelling individuals than in nursing home residents (*p* < 0.05). The daily intakes of sodium, potassium, magnesium, phosphorus, calcium, iron, zinc, and copper were significantly lower in community dwellers than in nursing home residents (*p* < 0.05). Selenium intake was also lower in nursing home residents, but the difference was not significant (*p* > 0.05).

**Table 3:** Participants’ energy, macro- and micronutrient intakes, and percentages meeting recommendations.

	Female						Male						Total	
	Nursing home			Community			Nursing home			Community			Nursing home	Community
	$\bar{X}\pm \mathrm{SD}$	Inadequate *n* (%)	Excessive *n* (%)	$\bar{X}\pm \mathrm{SD}$	Inadequate *n* (%)	Excessive*n* (%)	$\bar{X}\pm \mathrm{SD}$	Inadequate *n* (%)	Excessive *n* (%)	$\bar{X}\pm \mathrm{SD}$	Inadequate *n* (%)	Excessive *n* (%)	$\bar{X}\pm \mathrm{SD}$	$\bar{X}\pm \mathrm{SD}$
Energy (kkal)	1957.0 ± 279.0	—	1 (2.5)	1925.0 ± 340.3	—	3 (7.5)	2060.4 ± 342.5	—	4 (10.0)	2050.0 ± 379.4	—	3 (7.5)	2008.7 ± 314.7	1987.9 ± 363.5
CHO (g)	257.1 ± 52.8	—	2 (5.0)	254.9 ± 54.8	2 (5.0)	1 (2.5)	255.8 ± 44.9	3 (7.5)	—	271.4 ± 59.2	1 (2.5)	2 (5.0)	256.5 ± 48.7	263.1 ± 57.3
CHO (%)	53.7 ± 6.0			54.3 ± 6.9			51.0 ± 6.1			54.3 ± 6.8^*^			52.3 ± 6.2	54.3 ± 6.8
Protein (g)	73.5 ± 11.3	—	12 (30.0)	64.0 ± 10.4^**^	—	6 (15.0)	76.8 ± 12.8	—	23 (57.5)	69.5 ± 12.7^*^	1 (2.5)	11 (27.5)	75.1 ± 12.1	66.8 ± 11.9^**^
Protein (%)	15.5 ± 2.			13.7 ± 1.6^**^			15.2 ± 1.8			13.9 ± 1.7^*^			15.3 ± 2.1	13.8 ± 1.6^**^
Fat (g)	67.6 ± 15.6	1 (2.5)	7 (17.5)	69.2 ± 20.8	3 (7.5)	5 (12.5)	78.7 ± 22.3	1 (2.5)	12 (30.0)	73.3 ± 21.8	1 (2.5)	8 (20)	73.16 ± 19.98	71.30 ± 21.36
Fat (%)	30.7 ± 5.7			31.8 ± 6.8			33.6 ± 5.9			31.6 ± 6.4			32.1 ± 6.0	31.7 ± 6.6
Omega 3 (g)	1.61 ± 0.67	27 (67.5)	1 (2.5)	1.48 ± 0.76^*^	28 (70.0)	3 (7.5)	1.86 ± 0.73	14 (35.0)	2 (5.0)	1.55 ± 1.05^**^	30 (75.0)	3 (7.5)	1.73 ± 0.70	1.52 ± 0.91^**^
Omega 6 (g)	9.17 ± 3.75	—	—	8.66 ± 3.77	—	—	10.02 ± 3.84	—	—	10.85 ± 5.16	—	—	9.5 ± 3.7	9.7 ± 4.6
Saturated fat ac. (g)	26.80 ± 7.21	—	—	29.54 ± 9.90	—	—	32.91 ± 11.1	—	—	29.01 ± 8.4	—	—	29.8 ± 9.8	29.2 ± 9.1
Fiber (g)	30.8 ± 6.6	—	6 (15.0)	22.2 ± 5.6^*^	6 (15.0)	1 (2.5)	29.1 ± 5.4	—	9 (22.5)	23.6 ± 6.8^*^	4 (10.0)	4 (10.0)	29.9 ± 6.0	22.9 ± 6.2^**^
Vit. A (μg)	1698.6 ± 1526.0	1 (2.5)	28 (70.0)	1026.3 ± 392.2^*^	—	19 (47.5)	1077.5 ± 464.0	—	15 (37.5)	1301.8 ± 1023.4	—	16 (40.0)	1388.1 ± 1163.4	1164.1 ± 782.4
Vit. B (μg)	11.1 ± 3.4	4 (10.0)	7 (17.5)	12.0 ± 5.4	4 (10.0)	6 (15.0)	10.9 ± 3.1	7 (17.5)	1 (2.5)	12.4 ± 5.0	8 (20.0)	5 (12.5)	11.0 ± 3.3	12.2 ± 5.2
B_1_ Vit. (mg)	1.07 ± 0.16	—	1 (2.5)	0.87 ± 0.21^**^	1 (2.5)	1 (2.5)	1.11 ± 0.16	—	2 (5.0)	0.94 ± 0.23	—	2 (5.0)	1.09 ± 0.16	0.91 ± 0.22^**^
Vit. B_2_ (mg)	1.52 ± 0.34	—	4 (10.0)	1.34 ± 0.21^*^	4 (10.0)	—	1.43 ± 0.23	1 (2.5)	—	1.38 ± 0.27	5 (15.0)	—	1.47 ± 0.29	1.36 ± 0.24^*^
Vit. B_6_ (mg)	1.63 ± 0.30	1 (2.5)	1 (2.5)	1.27 ± 0.33^*^	11 (27.5)	—	1.57 ± 0.27	1 (2.5)	—	1.29 ± 0.35^**^	14 (35.0)	—	1.60 ± 0.29	1.28 ± 0.34^**^
Vit. B_12_ (μg)	7.31 ± 6.51	1 (2.5)	13 (32.5)	4.17 ± 1.57^*^	1 (2.5)	5 (15.0)	5.19 ± 2.95	2 (5.0)	14 (35.0)	4.76 ± 3.49^*^	7 (17.5)	4 (10.0)	6.25 ± 5.13	4.46 ± 2.71^**^
Folate (μg)	447.3 ± 104.0	—	19 (47.5)	296.9 ± 84.9^**^	7 (17.5)	3 (7.5)	385.0 ± 82.7	—	11 (27.5)	338.7 ± 105.5^*^	4 (10.0)	5 (15.0)	416.1 ± 98.5	317.8 ± 97.5^**^
Vit. C (mg)	164.8 ± 53.4	1 (2.5)	28 (70.0)	166.9 ± 58.2	—	25 (62.5)	143.0 ± 48.7	—	14 (35.0)	125.8 ± 45.1^*^	6 (15.0)	12 (30.0)	153.9 ± 51.9	146.3 ± 55.7
Sodium (mg)	3507.5 ± 547.0	—	33 (82.5)	3113.3 ± 756.4^*^	—	23 (57.5)	3787.6 ± 655.3	—	30 (75.0)	3143.4 ± 690.6	—	24 (60.0)	3647.6 ± 616.1	3128.4 ± 719.8^**^
Potassium (mg)	2736.9 ± 435.6	5 (12.5)	—	2407.9 ± 479.5^*^	13 (32.5)	—	2692.8 ± 330.3	5 (15.0)	—	2533.8 ± 555.4	10 (25)	—	2714.9 ± 384.7	2470.8 ± 519.4^**^
Calcium (mg)	811.4 ± 138.0	1 (2.5)	1 (2.5)	782.1 ± 165.6	7 (17.5)	—	827.9 ± 146.1	2 (5.0)	—	780.1 ± 150.4	2 (5.0)	—	819.7 ± 141.5	781.15 ± 157.2^*^
Magnesium (mg)	354.7 ± 55.2	—	3 (7.5)	272.4 ± 67.1^**^	2 (5)	2 (5.0)	347.5 ± 50.3	—	1 (2.5)	296.1 ± 72.5	7 (17.5)	1 (2.5)	351.1 ± 52.6	284.2 ± 70.4^**^
Phosphorus (mg)	1355.0 ± 217.0	—	40 (100.0)	1060.5 ± 206.5^**^	—	2 (5.0)	1360.91 ± 198.5	—	40 (100.0)	1137.3 ± 225.3	1 (2.5)	38 (95.0)	1358.0 ± 206.6	1098.9 ± 218.1^**^
Fe (mg)	13.08 ± 2.79	—	9 (22.5)	9.64 ± 2.66^**^	7 (17.5)	2 (5.0)	12.77 ± 1.88	—	7 (17.5)	11.05 ± 3.09	1 (2.5)	4 (10.0)	12.93 ± 2.37	10.35 ± 2.95^**^
Zn (mg)	14.07 ± 3.65	—	12 (30.0)	9.34 ± 2.02^**^	3 (7.5)	1 (2.5)	13.39 ± 2.67	—	2 (5.0)	10.14 ± 2.22^*^	8 (20.0)	—	13.73 ± 3.20	9.74 ± 2.14^**^
Cu (mg)	1.94 ± 0.49	—	20 (50.0)	1.45 ± 0.40^**^	—	8 (20.0)	1.74 ± 0.35	—	7 (17.5)	1.65 ± 0.50	2 (5.0)	5 (12.5)	1.84 ± 0.43	1.55 ± 0.46^**^
Selenium (μg)	16.52 ± 18.11	35 (87.5)	—	14.63 ± 14.24	37 (92.5)	—	16.66 ± 18.67	37 (92.5)	—	10.63 ± 9.41	39 (97.5)	—	16.59 ± 18.27	12.63 ± 12.16

^*^p < 0.05 and ^**^p < 0.01.

**Figure 2:**
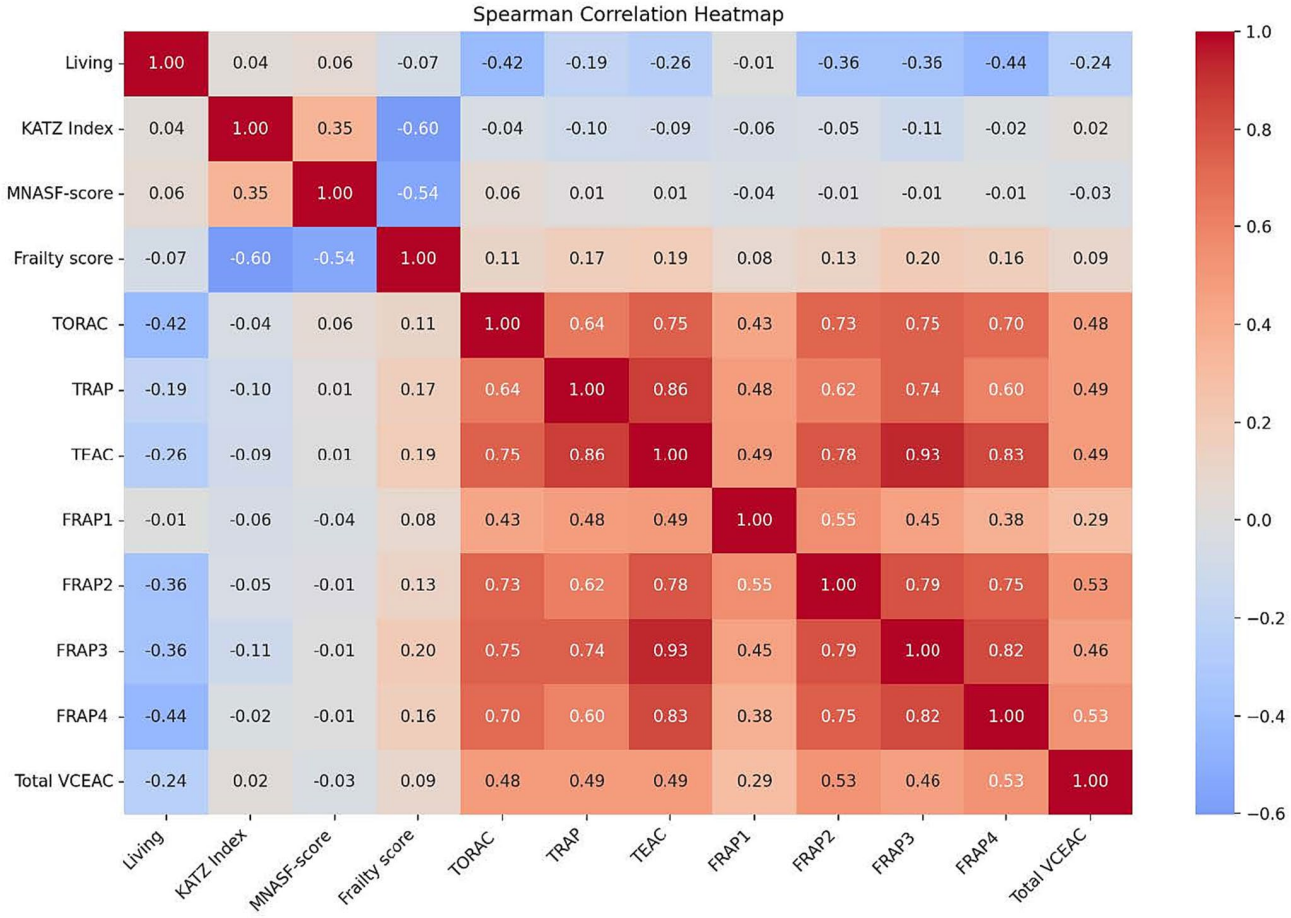
Association between KATZ, MNA-SF, frailty, and DTAC variables according to place of residence and frailty status.

Figure 2 shows the relationship between KATZ, MNA-SF, frailty, and DTAC variables according to place of residence and frailty status. The participants’ TORAC (*r* = −0.424, *p* < 0.001), TRAP (*r* = −0.190, *p* < 0.001), TEAC (*r* = −0.257, *p* < 0.001), and Total VCEAC (*r* = −0.241, *p* = 0.002) values showed a negative correlation with residential setting (reference: community). The KATZ Index (*r* = 0.036, *p* = 0.652), MNA-SF score (*r* = 0.061, *p* = 0.438), and frailty score (*r* = −0.068, *p* = 0.393) did not show a significant correlation with place of residence. The KATZ Index (*r* = −0.56, *p* < 0.001) and MNA-SF score (*r* = −0.44, *p* < 0.001) were strongly and negatively correlated with frailty, whereas TRAP (*r* = 0.21, *p* = 0.008), TEAC (*r* = 0.19, *p* = 0.016), and FRAP3 (*r* = 0.17, *p* = 0.028) showed weak positive correlations with frailty. FRAP1, FRAP2, FRAP4, TORAC, and Total VCEAC showed very weak correlations with frailty, which were not statistically significant.

## 4 Discussion

This study aimed to examine the relationship between DTAC and frailty in individuals over the age of 65 living in either nursing homes or the community, as well as to assess the impact of residential setting on this relationship. The study was conducted with 160 participants (50.0% nursing home residents, 50.0% female). Participants living in the community had lower rates of chronic disease, medication use, body weight (kg), BMI (kg/m^2^), waist circumference (cm), hip circumference (cm), body fat (%), and obesity but higher total muscle mass (kg), waist/hip ratio, and mild obesity than those living in nursing homes. The frailty and malnutrition rates were lower in the community dwellers than in those living in nursing homes. Energy (kcal), carbohydrate (g), protein (g), fat (g), fiber (g), micronutrient, and DTAC intakes were higher in nursing home residents than in community dwellers.

Older adults have a high prevalence of chronic diseases and a high likelihood of having multiple chronic conditions (37). Studies have reported that the number of comorbidities, medication use, body weight, BMI, and WC values are higher in older adults living in nursing homes compared to their peers in the community (37–39). The prevalence of chronic diseases among older adults living in nursing homes is generally higher than among their community-dwelling peers (40). Lober et al. (38) reported that 85% of older adults (both community and nursing home residents) had at least one chronic disease, while the prevalence of two or more chronic diseases was 38% among community dwellers and 31% among nursing home residents. Findings from a comparative study indicated that 77% of older adults living in the community had two or fewer comorbidities, whereas nursing home residents had higher rates of complex health conditions (41). In our study, individuals living in nursing homes had a higher prevalence of comorbid chronic diseases (91.3%) compared to their community-dwelling peers (78.7%). The existing health status of elderly individuals admitted to nursing homes, combined with factors such as limited freedom of movement and social isolation caused by institutional life, may paradoxically lead to a decline in their general health status.

In the current study, participants living in nursing homes had higher body weight, BMI, WC, HC, and body fat percentage (%) but lower WHR and total muscle mass (kg) compared to their community-dwelling peers. Furthermore, the rate of mild obesity was lower, while the rate of obesity was higher among individuals living in nursing homes compared to those in the community. These findings are consistent with previous studies. A study conducted in Iran reported that BMI, central obesity, and mid-arm circumference (MAC) measurements were higher in nursing home residents than in elderly community dwellers (42). On the other hand, Pavlovic et al. (43) reported that body weight, BMI, WC, HC, and MAC values were higher in community dwellers than in nursing home residents. Similarly, a study conducted in Türkiye by Yılmaz et al. (44) found that body weight and BMI were higher in individuals living in the community compared to those in nursing homes. These differences may be attributed to variations in anthropometric measurement methods, differences in nursing home admission criteria across countries, and the overall health status of older adults. Additionally, community dwellers have greater opportunities for physical activity, such as housework, shopping, walking, and outdoor activities, which may contribute to a healthier body composition profile.

Chronic diseases and multimorbidity are common issues among older adults and increase the risk of frailty by impairing physiological functions (45). Studies investigating the prevalence of frailty in older adults living in nursing homes and in the community have reported a wide variation, ranging from 10 to 80.1% in nursing home residents and from 3.8 to 70.6% in community dwellers (37). Lorber et al. (38) reported a frailty prevalence of 36.5% among elderly community dwellers and 58.5% among nursing home residents. Similarly, a study involving Polish older adults reported that the prevalence of frailty and pre-frailty in nursing home residents was 47.8 and 41.1%, respectively, while these rates were 4.5 and 27.2% among elderly community dwellers. Consistent with previous studies, we found the prevalence of frailty and pre-frailty was 36.2 and 36.2% among nursing home residents and 31.3 and 28.7% among elderly community dwellers, respectively (38, 46). We observed higher frailty rates in women living in the community and in nursing homes (35 and 40%, respectively) compared to men (27.5 and 32.5%, respectively); however, the difference was not statistically significant (*p* > 0.05). A study conducted in China among individuals over 60 years of age reported that the incidence of frailty syndrome was 60.6 per 1,000 person-years and increased with age, with a higher prevalence in women than in men (47). Women may be more prone to frailty due to their longer life expectancy and lower muscle mass compared to men.

The aging process, characterized by physiological, cognitive, emotional, and behavioral changes, negatively impacts nutritional status and increases the risk of malnutrition. Additionally, cognitive decline, depression, social isolation, and socioeconomic challenges further contribute to malnutrition (42). Studies have reported that individuals living in nursing homes may be more vulnerable to malnutrition (39, 43, 48). In a study conducted in Portugal, the prevalence of malnutrition and the risk of malnutrition among nursing home residents was 37.3%, whereas this rate was 11.2% among elderly community dwellers (48). Kolberg et al. (49) compared the prevalence of malnutrition among older adults receiving home care, living in nursing homes, and residing in the community in Norway. They found that malnutrition rates were significantly higher among home care recipients (26.4%) and nursing home residents (23.6%) compared to community dwellers (13.5%). In another study involving elderly individuals living in different settings, the proportion of individuals with a BMI <20 kg/m^2^ was reported to be 4.2% among community dwellers, 1.6–9% among geriatric day hospital patients, 4.5–9.4% among hospitalized patients, and 3.8–18.2% among nursing home residents (50). In our study, similar to previous research, the prevalence of malnutrition was higher in nursing home residents (21.3%) than in community dwellers (17.5%). This difference may be attributed to various interrelated factors, including dietary diversity, social interaction, overall health status, cognitive impairment, and physical frailty. Additionally, malnutrition rates at the time of admission to the nursing home may have also contributed to this difference between the two groups.

Losses in activities of daily living (ADL), functional capacity, and abilities with aging result in increased dependence over time. Factors such as advanced age, education level, gender, polypharmacy, marital status, place of residence, socioeconomic status, mental disorders, malnutrition, and chronic diseases influence this dependence (51, 52). In our study, similar mean ADL scores were observed in both groups, with values of 4.91 ± 1.38 in nursing home residents and 4.97 ± 1.36 in elderly community dwellers. The rates of dependency and partial dependency were 8.8 and 41.2%, respectively, among nursing home residents, compared to 8.8 and 37.5% among elderly community dwellers. Previous studies have also shown that individuals living in the community tend to have higher rates of independence (53). Consistently, a population-based study conducted in Bali reported ADL scores of 19.1 ± 1.8 for community dwellers and 12.1 ± 4.6 for nursing home residents (*p* = 0.000). Similarly, a study conducted in Türkiye reported that ADL scores, assessed using the Barthel Index, were higher in community dwellers compared to those living in nursing homes (92.2 ± 17.8 vs. 60.4 ± 30.1, *p* = 0.00) (54). Differences in dependency rates across studies may be due to various factors, including the ADL scale used, participants’ age, health status prior to nursing home admission, and sociodemographic characteristics.

Aging is associated with various health problems, including dysphagia, diarrhea, depression, dementia, and reduced motor function, which lead to the deterioration of nutritional status. The majority of elderly individuals consume various foods and nutrients at levels below the recommended intake (43, 55, 56). In Slovenia, the majority of nursing home residents (over 90%) had daily energy (1,637.4 kcal/day for men and 1,356.3 kcal/day for women) and protein (59.5 g for men and 51.4 g for women) intakes below the recommended values. In the same study, approximately 80% of the participants had a daily fat intake (mean 50.8 g/day, 32.5% of total energy intake) exceeding the recommended limits (55). Engelheart and Akner (56) found that elderly individuals living in nursing homes in Sweden had higher intakes of energy (kcal), carbohydrates (g/day and E%), fat (g/day and E%), saturated fatty acids (g/day), and cholesterol (mg/day), but lower intakes of protein (g/day and E%) and fiber (g/day) compared to those living in the community. In a study examining the daily nutrient intakes of individuals living in nursing homes and in the community in Iran, energy and protein intakes were found to be similar between the groups. However, the same study reported that nursing home residents had lower intakes of many micronutrients and saturated fats but higher intakes of polyunsaturated fats (42). In our study, no statistically significant difference was identified between the groups in terms of daily energy (kcal), carbohydrate (g), total fat (g), saturated fat (g), and omega-6 (g) intakes. However, the daily protein (75.1 ± 12.1 g vs. 66.8 ± 11.9 g), omega-3 (1.73 ± 0.70 g vs. 1.52 ± 0.91 g), and fiber (29.9 ± 6.0 g vs. 22.9 ± 6.2 g) intakes were significantly higher in nursing home residents compared to community dwellers. The assessment of micronutrient intakes revealed that nursing home residents had significantly higher intakes of vitamins A, B_1_, B_2_, B_6_, B_12_, C, and folate compared to community dwellers. Furthermore, the intakes of minerals, except for sodium, were also found to be higher in nursing home residents. The higher nutrient intake among nursing home residents may be attributed to several factors. Planned menus and consistent food availability may have enhanced dietary diversity, while assistance from care staff for individuals with physical or cognitive impairments may have facilitated adequate nutrient intake. Additionally, social interaction during meals in nursing homes may have contributed to better nutrition compared to eating alone. Regular monitoring of nutritional status enables the early detection of potential deficiencies and the implementation of necessary interventions. These factors may contribute to the better nutritional status observed in nursing home residents.

Nutritional problems, inadequate nutrient intake, and increased inflammation during the aging process play a significant role in the development of various health issues. A high DTAC is associated with reduced levels of inflammatory molecules (14, 18, 40, 57). In their study involving individuals in Türkiye, Yalçın (58) reported dietary TAC values of 241.7 ± 218.0 mg VCE, T-ORAC 13688.1 ± 3210.8 μmol TE, TRAP 3.67 ± 1.68 mmol TE, TEAC 3.58 ± 1.49 mmol TE, FRAP-1 3.42 ± 1.51 mmol, and FRAP-4 5.42 ± 3.8 mmol Fe^2+^. In their study examining the association between DTAC and frailty in Chinese older adults, Li et al. (14) found that high DTAC was negatively associated with pre-frailty [odds ratio (OR) = 0.66; CI: 0.52–0.84; *p* < 0.001] and frailty (OR = 0.71; CI: 0.50–0.1.03; *p* < 0.001). In a study conducted among elderly Japanese women, a high DTAC was reported to be associated with a reduced risk of frailty (18). In our study, TORAC (μmol TE/100 g), TRAP (mmol TE/100 g), FRAP1 (mmol Fe/100 g), FRAP2 (mmol Fe/100 g), FRAP3 (mmol Fe/100 g), FRAP4 (mmol Fe/100 g), TEAC (mmol TE/100 g), and total VCEAC de values were higher in nursing home residents than in community dwellers. Additionally, frailty showed a weak positive correlation with TRAP, TEAC, and FRAP-3. The higher daily intake of vitamins A, E, and C, as well as folate, iron, and selenium in nursing home residents compared to community dwellers, may have contributed to their higher DTAC.

## 5 Conclusion

This study examined the relationship between DTAC and frailty, as well as the impact of residential setting on this association, in individuals over 65 years of age residing in nursing homes and in the community. Compared to community dwellers, nursing home residents had higher rates of chronic diseases, medication use, body weight, BMI, waist circumference, and body fat percentage, while their muscle mass and waist-to-hip ratio were lower. Additionally, frailty and malnutrition rates were higher among nursing home residents. Although DTAC intake was similar in both groups, nursing home residents had higher DTAC values. Frailty showed a weak positive correlation with TRAP, TEAC, and FRAP3 values. Residential setting may influence health status, nutritional status, dietary total antioxidant capacity, and frailty in elderly individuals. In this context, promoting the consumption of foods rich in bioactive compounds such as polyphenols, as well as antioxidants and micronutrients, within the daily dietary intake of elderly individuals holds the potential to augment DTAC intake. For the elderly, dietary diversity is recommended to prevent frailty and malnutrition in nutrition counseling and menu planning. Further large-scale studies are needed to identify the specific factors contributing to these differences.

A key strength of this study is its inclusion of both community-dwelling and nursing home-dwelling older adults. The survey questions were designed to be easily understood and answered by participants. Additionally, the accurate collection of food consumption records by an experienced dietitian is another strength. However, this study has some limitations. These include its cross-sectional design, small sample size, and the fact that it was conducted in one of the most established and experienced nursing homes in Türkiye, as well as the lack of detailed information on participants’ pre-study health status and prior interventions. Among these limitations, the absence of a country-specific antioxidant database represents the most significant impediment. Furthermore, as participation was voluntary, the sample may not be fully representative of the baseline population characteristics. Finally, the study population was restricted to older adults of Turkish ethnicity; therefore, the findings need further validation across diverse ethnic and regional demographics to ensure generalizability.

## Data Availability

The raw data supporting the conclusions of this article will be made available by the authors, without undue reservation.
